# Influenza A Virus Hemagglutinin Trimer, Head and Stem Proteins Identify and Quantify Different Hemagglutinin-Specific B Cell Subsets in Humans

**DOI:** 10.3390/vaccines9070717

**Published:** 2021-07-02

**Authors:** Aafke Aartse, Dirk Eggink, Mathieu Claireaux, Sarah van Leeuwen, Petra Mooij, Willy M. Bogers, Rogier W. Sanders, Gerrit Koopman, Marit J. van Gils

**Affiliations:** 1 Department of Virology, Biomedical Primate Research Centre, 2288 GJ Rijswijk, The Netherlands; a.aartse@amsterdamumc.nl (A.A.); mooij@bprc.nl (P.M.); bogers@bprc.nl (W.M.B.); 2 Department of Medical Microbiology, Amsterdam Infection & Immunity Institute, Amsterdam UMC, University of Amsterdam, 1105 AZ Amsterdam, The Netherlands; w.d.eggink@amsterdamumc.nl (D.E.); m.a.claireaux@amsterdamumc.nl (M.C.); s.vanleeuwen1@amsterdamumc.nl (S.v.L.); r.w.sanders@amsterdamumc.nl (R.W.S.); 3 Department of Microbiology and Immunology, Weill Medical College of Cornell University, New York, NY 10021, USA

**Keywords:** antibody responses, influenza A virus, hemagglutinin, trimer specific, head, stem

## Abstract

Antibody responses against the influenza A virus hemagglutinin (HA)-protein are studied intensively because they can protect against (re)infection. Previous studies have focused on antibodies targeting the head or stem domains, while other possible specificities are often not taken into account. To study such specificities, we developed a diverse set of HA-domain proteins based on an H1N1_pdm2009_-like influenza virus strain, including monomeric head and trimeric stem domain, as well as the full HA-trimer. These proteins were used to study the B cell and antibody responses in six healthy human donors. A large proportion of HA-trimer B cells bound exclusively to HA-trimer probe (54–77%), while only 8–18% and 9–23% were able to recognize the stem or head probe, respectively. Monoclonal antibodies (mAbs) were isolated and three of these mAbs, targeting the different domains, were characterized in-depth to confirm the binding profile observed in flow cytometry. The head-directed mAb, targeting an epitope distinct from known head-specific mAbs, showed relatively broad H1N1 neutralization and the stem-directed mAb was able to broadly neutralize diverse H1N1 viruses. Moreover, we identified a trimer-directed mAb that did not compete with known head or stem domain specific mAbs, suggesting that it targets an unknown epitope or conformation of influenza virus’ HA. These observations indicate that the described method can characterize the diverse antibody response to HA and might be able to identify HA-specific B cells and antibodies with previously unknown specificities that could be relevant for vaccine design.

## 1. Introduction

The antibody responses elicited by influenza A virus infections or vaccines provide a key component in the protection against future influenza virus infections. Influenza virus hemagglutinin (HA) and neuraminidase (NA) are the most abundant viral glycoproteins on the surface of the virus and high levels of antibody response are elicited against HA [1,2,3]. HA is highly diverse; to date, eighteen subtypes have been described for both avian and human influenza A, with high genetic and antigenic diversity within each subtype [4,5,6]. This diversity is a challenge for the human immune system and it is therefore important to understand which domains are targets for (neutralizing) antibodies and what the conservation of these antibody targets is within and between different HA subtypes. This information is vital for the development of a vaccine that elicits long-lasting broadly protective responses.

The human antibody responses towards influenza A virus have been extensively evaluated over the years [7,8,9]. Antibodies towards the viral glycoprotein HA are divided into roughly two groups; antibodies that target the highly variable head domain and those that target the more conserved stem domain of HA. These two groups of antibodies have different binding epitopes, potency, mechanism of action and differ notably in binding and neutralization breadth. Antibodies elicited by conventional vaccines target primarily the immunodominant globular head domain of HA [10,11,12]. These antibodies generally bind the receptor binding site (RBS) and the adjacent (highly variable) antigenic sites [13,14] and they usually neutralize the virus by obstructing its binding to the receptor on the cell surface [15]. Although these antibodies are very potent, they are mostly subtype or even strain specific and exhibit limited cross-protection across different strains, let alone between different subtypes. As a consequence current vaccines are not able to cope with the evolving virus, in particular the variable head domain [16,17]. Therefore, new vaccine strategies are being developed to provide long-lasting and broad antibody responses targeting the more conserved HA stem domain [18,19]. Unlike head-specific antibodies, stem-specific antibodies do not block the attachment of the virus to the host cell but are blocking other steps of the viral replication cycle: stem-specific antibodies can affect conformational changes that are necessary for membrane fusion, HA maturation, or the release of novel viral particles [15,18]. While the HA head and stem domains feature prominently in antibody and vaccine studies, much less is known about other targets such as quaternary epitopes on the HA trimer.

Serological assays like hemagglutination inhibition assay (HAI) and enzyme-linked immunosorbent assay (ELISA) are used to investigate HA-specific antibody responses in sera. However, these assays are able to describe only the dominant target domain at a polyclonal antibody level. Isolating monoclonal antibodies (mAbs) provides detailed insight in targeted domains, potency and mechanism of action. In previous studies, stem or head domain binding antibodies were selected and further characterized, while other possible antibody binding profiles were not explored. To understand the total antibody response towards influenza A virus’ HA without pre-selecting head or stem domain specific antibodies, a full characterization of the antibody responses targeting different domains is needed and requires the analysis of mAbs generated from trimeric HA or subdomain-specific B cells.

Here, we describe a flow cytometry-based strategy for the quantification of HA-directed B cell specificities distinguishing those directed against the head domain, the stem domain and non-head/stem domain specificities. The findings and methodologies allow for the isolation and subsequent in-depth characterization of mAbs targeting specific domains, providing key information on antibody responses induced by natural influenza virus infection and/or immunization with licensed or experimental vaccines.

## 2. Materials and Methods

### 2.1. Healthy Donors and PBMC Isolation

Buffycoats containing plasma, red blood cells and Peripheral Blood Mononuclear Cells (PBMCs) of six different healthy donors were obtained from the Dutch bloodbank Sanquin (collected in March and June in 2019). To obtain the plasma of the same time point as the PBMCs, the buffycoats were centrifuged 10 min at 2000 rpm, the plasma was taken and centrifuged again for 10 min at 11,000 rpm to eliminate residual cell debris. PBMCs were isolated by density gradient centrifugation separation using LeucoSep tubes (Greiner, Alphen aan de Rijn, The Netherlands) for the separation of different cell layers, using Lymphoprep density gradient medium (StemCell technologies, Dieren, The Netherlands) according to manufacturer’s protocol. The cells were cryopreserved in 10% dimethyl sulfoxide/Fetal Calf Serum (DMSO/FCS) until further analysis.

### 2.2. Recombinant Protein Design, Production and Purification

**Figure 1:**
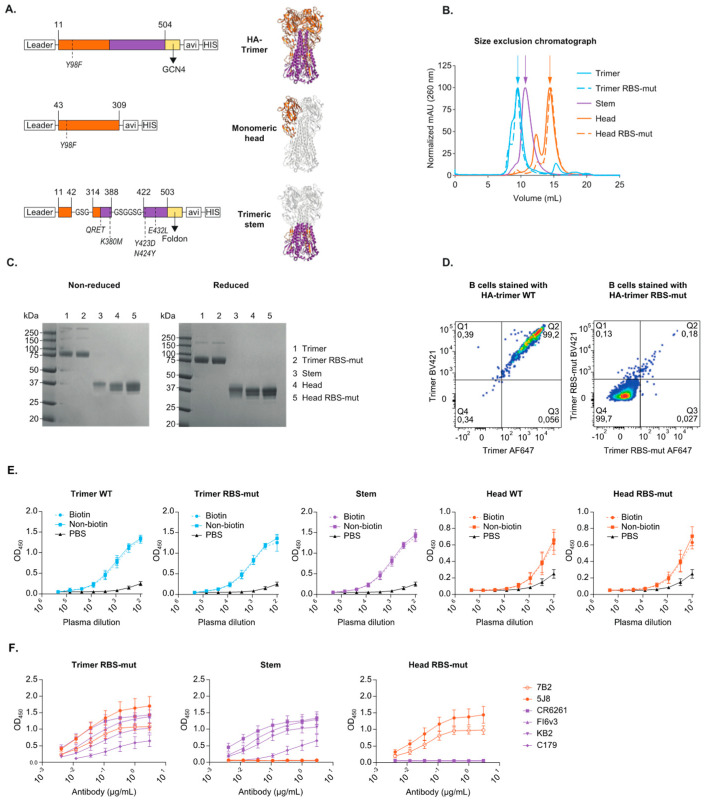
Design and characterization for of H1N1_pdm2009_ HA-trimer, trimeric stem and monomeric head probes. (**A**) A linear representation and crystal structure (PDB: 3LZG, adjusted with PyMol) of the HA-protein designs highlighting the mutations and modifications that were introduced (H3 numbering); a trimerization domain, an avi-tag and a his-tag. The HA-trimer can be cleaved into HA1 (orange) and HA2 (purple). The designed head-domain (orange) constitutes of parts of HA1 and stem-domain (purple) constitutes of parts of HA1 and HA2. (**B**) A size exclusion chromatograph (SEC) of the HA-trimer (blue), monomeric head (orange) and trimeric stem (purple) protein. The WT protein is the solid line and the RBS-mut is the dotted line. The arrows mark the expected peaks corresponding to the size of the proteins. (**C**) An SDS-PAGE analysis of the different HA-proteins under non-reducing (**left**) and reducing (**right**) conditions. (**D**) Flow cytometry plots gated on single, live, CD20+ and CD3- total B cells and stained with HA-trimer WT (**left**) or HA-trimer RBS-mut (**right**), both conjugated to both AF647 and BV421 for dual staining. (**E**) The polyclonal antibody binding profile to biotinylated protein (solid line) compared to non-biotinylated protein (dotted line) assed for both WT and RBS-mut proteins, tested in a Ni-NTA ELISA with plasma of a healthy donor. (**F**) mAb binding patterns for HA-trimer RBS-mut, trimeric stem and monomeric head RBS-mut proteins to examine the folding of the proteins in a Strep-Tactin XT ELISA. Stem-domain mAbs are in purple and head-domain mAbs are in orange.

The recombinant proteins of HA were designed based on the sequence of H1N1_pdm2009_ A/Netherlands/602/2009 (GenBank: CY039527.2). These proteins were termed HA-trimer, monomeric head and trimeric stem (Figure 1A). Soluble HA-trimer (residues 11-504, H3 numbering [20]) was constructed by replacing the viral transmembrane domain by the trimerization domain GCN4. The soluble stem protein design was previously described [21,22] and we converted this design into H1N1_pdm2009_ of A/Netherlands/602/2009. The original HA leader was used for both the HA-trimer and the trimeric stem designs. The monomeric head protein (residues 43-309, H3 numbering [20]) contained the tissue plasminogen activator (TPA) leader sequence instead of the original HA-leader. The previously described [23] receptor binding site mutation (RBS-mut) Y98F was introduced in both the HA-trimer and monomeric head proteins by Site-Directed Mutagenesis (QuickChange II, Agilent) according to the manufacturer’s protocol. All five recombinant proteins (HA-trimer, HA-trimer RBS-mut, monomeric head, monomeric head RBS-mut and trimeric stem) contained sequentially a C-terminal avi-tag and His_6_-tag for labelling and purification purposes respectively.

The genes encoding for the HA-trimer, monomeric head and trimeric stem proteins were ordered (GenScript, Leiden, The Netherlands) and cloned into the expression vector pPPI4 [24] by using Gibson assembly [25]. The Gibson assembly was carried out as described previously [26]. Briefly, the gene-DNA and the expression vector were added in a 1:1 ratio to the 2X home-made Gibson assembly mix. The genes and the introduced RBS-mut were verified by Sanger sequencing.

All proteins were expressed in a mammalian cell expression system as described previously [27] with some minor changes. In short, suspension HEK293F cells (Invitrogen, cat no. R79007) were cultured in FreeStyle medium (Gibco, ThermoFisher Scientific, Amsterdam, The Netherlands) at a density of 0.8–1.2 million cells/mL and were transfected with the plasmid expressing one of the five protein genes in a 1:3 ratio with 1 mg/mL PEImax (Polysciences Europe GmbH, Eppelheim, Germany). The supernatant was harvested five days after transfection, centrifuged 25–30 min at 4000 rpm and filtered using 0.22 µm pore size SteriTop filters (Millipore, Amsterdam, The Netherlands). Filtered supernatant was run over a nickel-agarose (Ni-NTA) beads column (Qiagen, Venlo, The Netherlands). The proteins were purified according to the manufacturer’s instructions (Qiagen, the QIAexpressionist 06/2003). The protein-elution was done with 10–30 mL of the described Elution Buffer.

The recombinant HA proteins were buffer exchanged to phosphate-buffered saline (PBS) and concentrated by use of 100 kDa (trimer), 50 kDa (stem), or 30 kDa (head) VivaSpin20 columns (Sartorius, Gӧttingen, Germany), followed by Size Exclusion Chromatography (SEC), using a SuperDex200 10/300 GL increase column. The peak-fractions corresponding to the HA-trimer, monomeric head, or trimeric stem were pooled, concentrated again and stored in PBS at −80 °C.

The protein concentration was determined on the NanoDrop 2000, using the theoretical molecular weight and the extinction coefficient calculated with Expasy (ProtParam-tool). The purified SEC-proteins were quality checked and characterized in a reduced (supplemented with 1 M DTT) and in a non-reduced condition on an SDS-PAGE using a 10–20% Tris-Glycine gel (Novex, Invitrogen, Amsterdam, The Netherlands) and stained with Coomassie blue.

The purified proteins were biotinylated in vitro with the BirA kit (GeneCopoeia, tebu-bio, Heerhugowaard, The Netherlands) using the same conditions for all proteins. Subsequently the HA proteins were buffer exchanged to PBS and stored at −80 °C.

### 2.3. HA-Trimer Specific Single B Cell Sorting by Flow Cytometry

HA-trimer specific B cell response from six healthy human donors was analyzed by flow cytometry. Biotinylated recombinant proteins were conjugated with streptavidin fluorophores resulting in fluorescent labelled-probes. HA-trimers, both WT and RBS-mut, trimeric-stem and monomeric-head were conjugated in an 8:1 ratio (*w*/*w*) to streptavidin-conjugates BV421 (0.1 mg/mL, BioLegend, Amsterdam, The Netherlands), BB515 (0.1 mg/mL, BD, Vianen, The Netherlands), or AF647 (0.5 mg/mL, BioLegend) at 4 °C, for 1–2 h.

PBMCs were thawed and stained with the following surface markers: CD3-v500 (UCHT1, BD), CD20-PE-CF594 (2H7, BD), IgM-BV605 (MHM-88, BioLegend), IgG-PE-Cy7 (G18-145, BD), IgA-PE (polyclonal, Southern Biotech), the live/dead marker (viability-eF780, Invitrogen) and streptavidin-conjugated probes at 4 °C, for 30–60 min. Flow cytometry was performed on an ARIA 4 lasers (BD) flow cytometer and the data analysis was performed with FlowJo v10.6.

Live, CD3-, CD20+ and HA-trimer+ cells were single cell sorted into a 96-well plate that contained 20 µL lysis buffer consisting of 20 U RNAse inhibitor (Invitrogen), first strand superscript III buffer (Invitrogen) and 1.25 µL of 0.1 M DTT (Invitrogen). The plates with the sorted cells were stored at −80 °C for at least 24 h before performing the RT-PCR to obtain cDNA.

### 2.4. Generation of Antigen Specific Antibodies

An RT-PCR was performed to reverse transcribe the RNA of the single B cells to cDNA. Briefly, 50 U SuperScript III RTase (Invitrogen), 2 µL of 6 mM dNTPs (Invitrogen) and 200 ng random hexamer primers (Invitrogen) in a total volume of 6 µL was added to the plate containing the sorted cells and lysis buffer. The RT program was set as followed: 10 min at 42 °C, 10 min 25 °C, 60 min at 50 °C, 5 min at 95 °C and infinity 4 °C. The cDNA was stored at −20 °C until further analysis.

The V(D)J variable regions of the antibodies were amplified as described previously [26,28,29,30]. The amplified variable V(D)J-region of the heavy and light chain of the antibody were cloned into correspondingly expression vector containing the constant regions of the human IgG for the heavy or light chain by Gibson Assembly and were checked by Sanger sequencing. Sequences were uploaded into the IMGT-database (V-QUEST) to obtain the CDR3-properties and V-usages.

### 2.5. Recombinant Antibody Production

The recombinant antibodies were first tested in a small-scale experiment as described previously [26], with Lipofectamine 2000 (Invitrogen) as transfection reagent in 1:21 ratio in a final volume of 50 µL, the supernatant was harvested after 72 h and were tested in an IgG-coated enzyme-linked immunosorbent assay (ELISA). The previously discovered monoclonal antibodies 5J8 [31,32], CR6261 [33,34], FluA-20 [35], CR8020 [36,37] and FI6v3 [38] and the newly discovered recombinant antibodies from this study were produced in a mammalian HEK293F expression system. Briefly, suspension HEK293F cells (Invitrogen, cat no. R79007) at a density of 0.8–1.2 million cells/mL were co-transfected with the two plasmids expressing the heavy and light chains (1:1 ratio) in a 1:4 ratio with 1 mg/mL PEImax (Polysciences). The recombinant IgG antibodies were purified from the supernatant after five days as described previously [39]. In short, the supernatant was filtered with 0.22 µm pore size SteriTop filters (Millipore) and run over a protein G agarose packed column (ThermoScientific, Amsterdam, The Netherlands) followed by two column volumes of PBS wash. The antibodies were eluted with 0.1 M glycine pH 2.5, into the neutralization buffer 1 M TRIS pH 8.7 in a 9:1 ratio. The purified antibodies were buffer exchange to PBS using 100 kDa VivaSpin20 columns (Sartorius). The IgG concentration was determined on the NanoDrop 2000 (ThermoScientific) and the antibodies were stored at 4 °C. The stem antibody C179 [40] was purchased from Takara and 7B2 and KB2 were isolated as described previously [41,42,43].

### 2.6. Binding of Monoclonal Antibodies and Plasma in Enzyme-Linked Immunosorbent Assay (ELISA)

Two different ELISAs were performed to check the binding of the monoclonal antibodies and plasma to the recombinant HA proteins; pre-coated Ni-NTA (HisSorb, Qiagen) or Strep-Tactin XT (iba Lifesciences GmbH, Gӧttingen, Germany) ELISA plates. The recombinant proteins were coated at a 1 µg/mL final concentration for 2 h, followed by a washing step with TBS and residual blocking of 30–60 min with 2% skimmed milk in TBS (*w*/*v*). After a TBS wash, the primary antibody (starting at 3 µg/mL antibody or 1:100 diluted plasma) was three-fold serially diluted and incubated for 2 h, followed by a TBS wash. The secondary antibody labelled with horseradish peroxidase (HRP) was diluted 1:3000 or 1:10,000 (goat-anti-mouse in 0.1 mg/mL or goat-anti-human in 1 mg/mL respectively (KPL antibodies & conjugates)) and incubated for 1 h. Next, a washing step was performed with TBS/Tween-0.05%, the substrate supplemented with 1% TMB and 0.01% hydrogen peroxide was added, and the color development was stopped with 0.8 M sulfuric acid. The readout was done using a SPECTROstar Nano plate reader (BMG Labtech, De Meern, The Netherlands) and the OD was measured at 450 nm. All ELISAs were performed in duplicate, on two independent days (*n* = 4).

### 2.7. Viruses

Four different virus strains were used in hemagglutination inhibition (HAI) and micro neutralization (MN) assays. These viruses include pre-H1N1_pdm2009_ strain (A/Netherlands/02/2003), H1N1_pdm2009_-like strain (A/Netherlands/602/2009), post-H1N1_pdm2009_ strain (A/Netherlands/10218/18) and 6:2 recombinant H5N1 strain (A/Indonesia/5/2005, in a PR8-backbone). Viruses were propagated on Madin-Darby Canine Kidney (MDCK) cells in infection medium consisting of MEM-Eagle Medium EBSS (Lonza, Geleen, The Netherlands) supplemented with MEM Non-Essential Amino Acids (Gibco), penicillin (100 U/mL), streptomycin (100 µg/mL), L-Glutamine (Lonza), HEPES (Lonza), and TPCK-trypsin (Sigma-Aldrich/Merck, Darmstadt, Germany). After 72 h of incubation viruses were harvested. Virus titers (fifty-percent tissue culture infective dose (TCID50)) was determined as described [44]. In short, MDCK cells were inoculated with 10-fold serial dilutions of virus stocks and incubated at 37⁰C/5% CO_2_. Three days after inoculation, supernatants of cell cultures were tested for agglutinating activity using turkey red blood cells (tRBCs) as an indicator of virus replication. Infectious virus titers were calculated by the method of Reed and Muench [45,46].

### 2.8. Hemagglutination Inhibition (HAI) Assay

The hemagglutination inhibition activity of the recombinant antibodies and the control antibodies 7B2, CR9114, 5J8 and FluA-20 was tested in duplicate in an HAI-assay. Hemagglutination titers of viruses were determined and virus stocks were diluted to a concentration of 4 hemagglutinin units (HAU) as described previously [46]. In short, antibodies were two-fold serially diluted in PBS starting from 2 µg/mL for suspected head antibodies and 100 µg/mL for suspected stem or trimer antibodies. Viruses were incubated with serially diluted antibodies in a total volume of 75 µL for 30 min at 37 °C. A total of 25 µL 1% of tRBCs was applied on top of the complexed antibody and virus. This resulted in 0.25% of tRBCs and a final starting antibody concentration of 1 µg/mL for suspected head antibodies and 50 µg/mL for suspected stem or trimer antibodies. Hemagglutination patterns were scored after one h of incubation at 4 °C.

### 2.9. Micro-Neutralization (MN) Assay

The neutralization capacity of the recombinant antibodies and the control antibodies 7B2, CR6261, 5J8 and FluA-20 was assessed in triplicate by an MN-assay. In short, MDCK cells were cultured one day prior MN-assay in a 96-well plate to obtain 90–100% confluency. The antibodies were 3-fold diluted in infection medium and pre-complexed with the viruses in a concentration of 2000 TCID/mL for 2–2.5 h at 37 °C. The MDCK cells were washed once with PBS, the virus and antibody complexes were applied on the cells and incubated for 2–2.5 h at 37 °C. The cells were washed again once with PBS and incubated for 48 h at 37 °C with the corresponding antibody dilutions in the overlay. After 48 h, the cells were washed with PBS, fixated with ice-cold 80% acetone and stored at −20 °C. The uptake of the viruses by the MDCK-cells was assessed by ELISA using anti-NP antibody HB65 (anti-mouse) in a 1:3000 dilution. The average signal was taken from triplicates, normalized to the virus control (0% neutralization) and cell control (100% neutralization) and the 50% inhibitory concentration (IC50) of each antibody was calculated using GraphPad Prism 8, or set at the maximum antibody concentration tested when no neutralization was observed.

### 2.10. Competition Assay with Monoclonal Antibodies

Previously characterized mAbs were used in a competition assay by use of optical biosensor platform Octet (Sartorius) to assess the binding epitope of the isolated mAbs. First, a baseline was obtained by dipping the Ni-NTA biosensor (Sartorius) in running buffer (0.1% BSA and 0.01% Tween-20 in PBS) for 60 s. The non-biotinylated HA-trimer RBS-mut protein (10 µg/mL) was immobilized onto the biosensor tips for 240 s. The baseline signal was measured again for 60 s before dipping the biosensor into the wells containing the primary antibody (5 µg/mL) for 120 or 240 s. After this step, the sensor was dipped into the wells with the secondary antibody (5 µg/mL) for 120 s. Residual binding was determined by dividing the maximum binding of the secondary mAb in presence of the primary mAb by the maximum binding of the secondary mAb without competitor, multiplied by 100. Non-competing mAbs had a value of ≥66, intermediate competition was scored between 33 and 66 and competition was scored for ≤33.

## 3. Results

### 3.1. Design and Characterization of Recombinant HA-Trimer, Monomeric Head and Trimeric Stem Probes

In order to study domain specific antibody and B cell responses against influenza virus’ HA in humans, we designed and characterized three recombinant HA-protein constructs. These soluble proteins included HA-trimer, monomeric head domain and trimeric stem domain and were based on H1N1_pdm2009_-like influenza virus strain A/Netherlands/602/2009 (Figure 1A). The HA-trimer and monomeric head proteins were designed without (wild type, WT) and with the receptor binding site-mutation (RBS-mut) Y98F [23] (H3 numbering [20]).

The purity of the recombinant proteins after production and purification was examined by size exclusion chromatography (SEC) (Figure 1B) and SDS-PAGE using Coomassie blue staining (Figure 1C). Some minor impurities were observed in the SEC-profile and appropriate fractions were pooled and concentrated for further use and analyses. A non-reduced and reduced SDS-PAGE stained with Coomassie blue was performed and all proteins showed the expected size on the gels; full-length HA-trimer around 75 kDa, monomeric head and trimeric stem around 30 kDa and no significant impurities were observed. The different bands for the monomeric head proteins on the SDS-PAGE can likely be explained by the differences in glycosylation and glycan occupancy of the protein [47,48].

Influenza virus uses sialic acids (SIAs) on the target cells as its attachment receptor and is able to bind to these cellular surface SIAs via the RBS of HA [49]. The Y98F substitution [23] in HA was reported to disrupt this binding without affecting the binding of RBS-directed antibodies [50]. Indeed, we observed that the WT-trimer probe (Figure 1D, left) bound to all B cells (CD20+/CD3-) while the RBS-mut trimer probe (Figure 1D, right) only bound to a small subset of B cells, most likely in an antigen-specific manner. Hence, the RBS-mut is necessary for the H1N1_pdm2009_ HA to circumvent nonspecific binding and reduce the major background staining of B cells to the probes of interest. Therefore, only the RBS-mut variant of the HA-trimer and monomeric head proteins were used in further analyses.

Next, the avi-tags of the proteins were covalently bound to biotin before the protein was conjugated to a streptavidin fluorophore to create a fluorescent probe. The biotinylation process did not affect the binding of polyclonal plasma from a human healthy donor as shown by enzyme-linked immunosorbent assay (ELISA) (Figure 1E). The three final protein constructs (biotinylated HA-trimer RBS-mut, monomeric head RBS-mut and trimeric stem) were also characterized in more detail using monoclonal antibodies (mAbs) to confirm the correct folding of the proteins (Figure 1F and Figure S1). The broadly reactive and stem-specific mAbs CR6261 [33,34], C179 [40], KB2 [42,43] and FI6v3 [38] bound to both the HA-trimer RBS-mut and trimeric stem, but not the monomeric head RBS-mut. The H1N1_pdm2009_ head-specific and RBS-directed mAbs 7B2 [41] and 5J8 [31,32] were able to bind both HA-trimer and monomeric head RBS-mut, while no binding was observed for the trimeric stem protein as expected. As well the trimer interface mAb FluA-20 [35] was able to bind the monomeric head RBS-mut protein, while no binding was observed for the trimeric stem protein (Figures S1 and S2). The head-specific mAb 5J8 binds to the receptor binding domain with a long CDRH3-chain that is entering into the binding pocket, mimicking a SIA. 5J8 was still able to bind to the RBS-mut protein variants, which indicates that the RBS-substitution does not interrupt the binding of such RBS-specific antibodies, although it is disrupting the binding to cell surface SIAs. The binding of the broadly reactive, well characterized mAbs to the biotinylated HA-trimer RBS-mut, monomeric head RBS-mut and trimeric stem proteins confirmed that these proteins are suitable for the identification of influenza virus HA-specific B cells.

### 3.2. HA-Trimer, Monomeric Head and Trimeric Stem Probes Identify and Quantify Distinct HA-Specific B Cell Subsets

**Figure 2:**
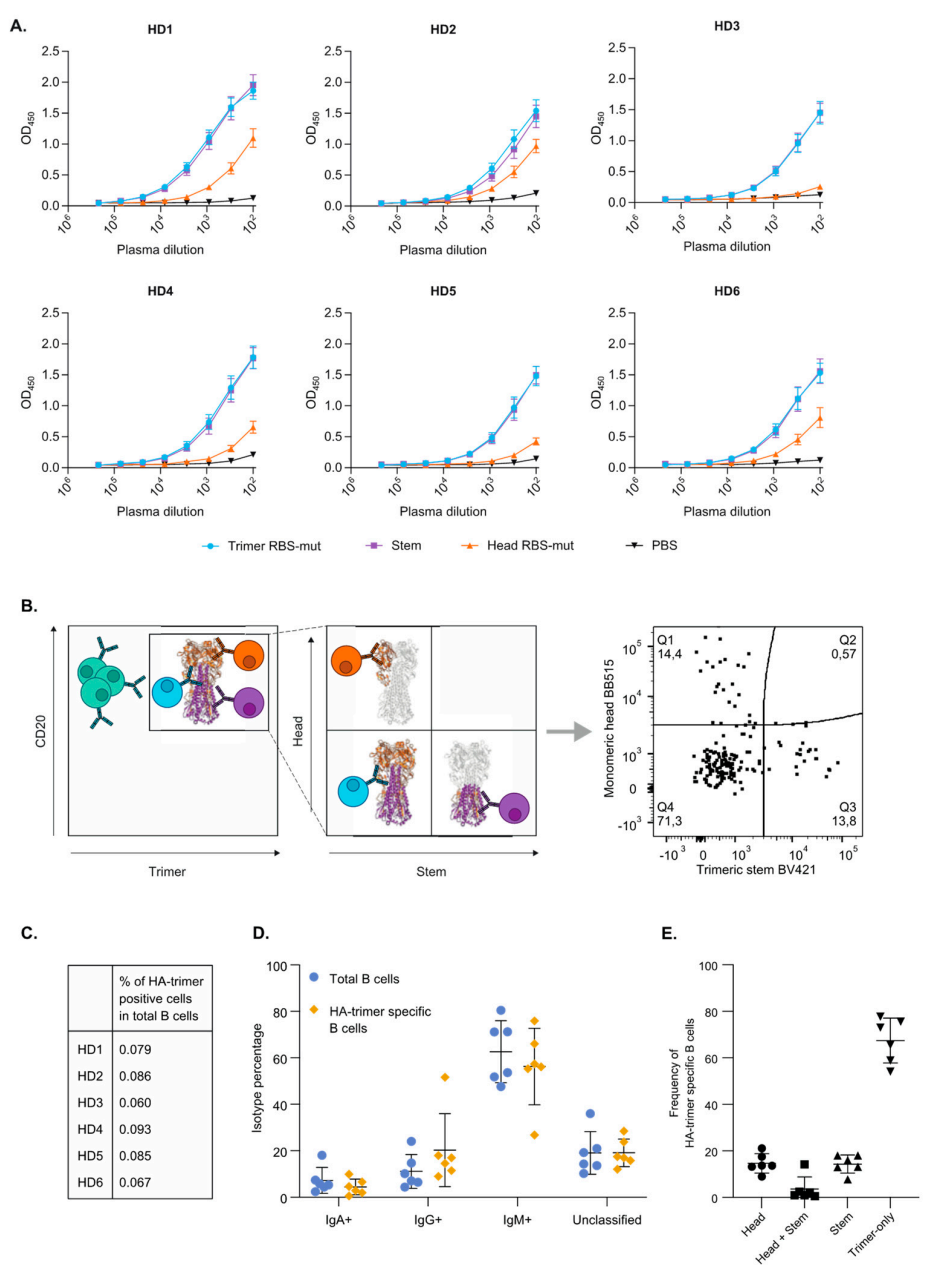
Binding domains and isotypes of HA-trimer H1N1_pdm2009_ specific B cells. The antibody responses of six healthy donors were evaluated on antibody level by ELISA and on B cell level by flow cytometry. (**A**) The IgG antibody responses in plasma of six healthy donors (HD) to HA-trimer RBS-mut (blue), trimeric stem (purple), or monomeric head RBS-mut (orange) were measured in a Strep-Tactin XT ELISA. (**B**) A schematic representation of the triple-probe flow cytometry strategy used in this study. The HA-trimer specific cells (CD20+/HA-trimer+) are depicted in the left panel together with HA-negative B cells (in green). The HA-trimer specific population was analyzed as shown in the right panel together with representative flow cytometry plot, with monomeric head+ cells (orange), trimeric stem+ cells (purple) and the cells that are not recognizing the head or stem probe (blue). (**C**) Frequencies of HA-trimer specific B cells among total B cells in six healthy donors. (**D**) Frequencies of IgA, IgM and IgG positive B cells among total B cells (blue) and HA-trimer specific B cells (yellow). B cells that were double/triple positive or negative for all three isotypes were unclassified. (**E**) Frequencies of probe positive cells among the population of HA-trimer specific B cells.

The H1N1_pdm2009_ constructs were used as probes to quantify the proportion of HA-specific B cells of six human healthy donors sampled in 2019 and determine the distribution of head-specific and stem-specific B cells. First, the plasma of these six donors were tested in ELISA to measure the polyclonal antibody response against the different H1N1_pdm2009_ proteins (Figure 2A and Figure S2). All donors showed a highly similar antibody response against the H1N1_pdm2009_–like influenza virus strain with both stem and head-specific antibodies present. The stem-specific responses were of similar magnitude as the response against the HA-trimer, while the head-specific responses were weaker. In order to study whether a similar pattern could be found in the B cell population of these donors, the peripheral blood mononuclear cells (PBMCs) were stained with surface markers and the designed probes to identify the HA-specific B cells (CD20+/CD3- cells) (Figure 2B and Figure S3).

The frequencies of the HA-trimer specific B cells were highly similar between all donors tested and varied between 0.06% and 0.09% of the total B cell population (Figure 2C). Combined staining for IgM, IgG and IgA showed that the majority of the HA trimer specific cells were IgM positive (27–76%), while lower frequencies of IgG+ and IgA+ HA-trimer specific B cells were observed (9–52% and 1–10% respectively). This pattern was consistent across all six donors with only modest differences between individuals (Figure 2D and Figure S4). While frequencies of IgA+ HA-trimer specific B cells were similar as in the frequencies of IgA+ cells total B cell population, HA-trimer specific B cells were slightly enriched in IgG-class switched B cells compared to the total B cells, resulting in a relative minor decrease of IgM+ B cells in the HA-trimer specific population.

Next, the binding domains of the HA-trimer specific B cells were analyzed as illustrated schematically in Figure 2B. While both head and stem specific B cells were identified within the HA-trimer specific B cell population, these constituted minorities (9–23% and 8–18%, respectively; Figure 2E). Remarkably, the majority of HA-trimer specific B cells that did not recognize the monomeric head nor the trimeric stem and was designated as trimer-only specific (54–77%; Figure 2E). To exclude an influence of the selected fluorophores, cells of two healthy donors were stained with viability, CD3 and CD19 surface markers and HA-trimer labelled with different fluorophores. The outcome was the same and revealed a similar distribution of head, stem and trimer-only specific B cells (Figure 2B and Figure S5).

### 3.3. Monoclonal Antibodies Confirm the Head, Stem and Trimer-Only Specificities

**Figure 3:**
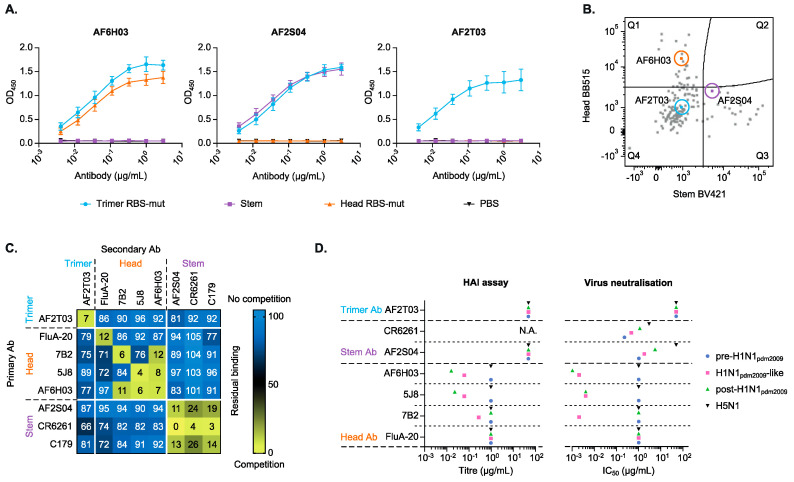
Binding domain and functional analysis of HA-trimer, head and stem specific mAbs. (**A**) The antibody binding patterns of the three mAbs in Strep-Tactin XT ELISA coated with the HA-trimer (blue), monomeric head (orange) and trimeric stem (purple). (**B**) Index sorting showing the flow cytometry plot highlighting the B cells that yielded the three mAbs studied further. The plot shows merged staining profiles of sorted trimer specific B cells of two HDs. (**C**) A heatmap of a competition assay with well characterized mAbs and the newly discovered mAbs. The values represent the residual binding of secondary antibody in combination with the primary antibody after pre-incubation of the HA-trimer protein, ranging from no competition (blue) to (strong) competition (yellow). (**D**) The antibodies were tested in HAI (left panel) and virus neutralization (right panel) assays against the autologous H1N1 virus, two heterologous H1N1 viruses and an H5N1 virus. The antibodies are grouped according to their binding domain. When no neutralization or HAI was observed the IC50 or titer were set at 50 µg/mL for trimer and stem specific antibodies and 1 µg/mL for head specific antibodies. CR6261 was not tested in HAI (N.A.).

HA-trimer, head and stem specific B cells from two donors (HD2 and HD6) were single cell sorted. The variable genes of the antibody heavy and light chain were amplified and cloned into expression vectors for secreted IgG. Eight heavy and light chain pairs were selected and screened for their expression and binding properties (Figure S6, left). Seven of these eight pairs yielded detectable IgG and six out of these seven IgG antibodies reacted with the HA-trimer protein (Figure S6, right). Three antibodies, including one derived from a head-specific B cell (AF6H03), one from a stem-specific B cell (AF2S04) and one from a trimer-only B cell (AF2T03) were selected for in-depth characterization. While these antibodies were all expressed as IgG, the original isotypes were IgG for the stem antibody AF2S04, IgA for the trimer-only antibody AF2T03 and unknown for the head antibody AF6H03 (i.e., the original B cell stained negative for IgG, IgA and IgM). The binding patterns of the three purified antibodies in ELISA (Figure 3A) were consistent with the flow cytometry staining of the original derived B cells (Figure 3B). Thus, antibody AF6H03 bound to the head domain, antibody AF2S04 to the stem domain and AF2T03 only showed binding to the trimer. mAb sequence information such as CDR3-length and V-usage was obtained via IMGT/V-QUEST (Table S1). As of for several known stem antibodies [18], AF2S04 stem mAb uses VH1-69, while AF2T03 uses VH3-30 and mAb AF6H03 uses VH3-30-3 with the largest CDR3-length of the three isolated mAbs.

The binding domain of the mAbs was examined in more detail by use of a competition assay with previously published mAbs (Figure 3C). Head specific mAb AF6H03 competed with two known head specific mAbs 7B2 and 5J8, suggesting that the AF6H03 epitope overlaps with the epitopes of 7B2 and 5J8. Remarkably, mAbs 7B2 and 5J8 do not cross compete, suggesting that mAb AF6H03 occupies an epitope in between those of 5J8 and 7B2. The stem specific mAb AF2S04 showed competition with the known stem mAbs C179 [40] and CR6261, suggesting the epitope of AF2S04 overlaps the epitopes of these mAbs. Our trimer-only mAb AF2T03 did not compete with any tested stem or head specific mAbs, nor the head-interface mAb FluA-20, suggesting that AF2T03 binds to a unique as yet unidentified epitope. AF2T03 does not bind to the GCN4-trimerization domain or to the avi/biotin and his-tag (Figure S7).

Next, functional assays were performed to examine the agglutination inhibition and neutralization capacity of the antibodies. In general, head antibodies are inhibitory in a hemagglutination inhibition (HAI) assay, while stem antibodies do not have this agglutination inhibition activity. As expected, the head antibody AF6H03 did show HAI activity against both H1N1_pdm2009_-like virus and post-H1N1_pdm2009_ virus, while the stem antibody AF2S04 did not (Figure 3D, left panel). The HAI activity of AF6H03 against these two viruses was similar to that of head-specific mAb 5J8. Head-specific mAb 7B2 did inhibit H1N1_pdm2009_ virus but failed to inhibit the post-H1N1_pdm2009_ virus, suggesting that AF6H03 antibody targets a more conserved domain than mAb 7B2. Interestingly, the trimer-only antibody AF2T03 also did not show HAI activity (>50 µg/mL), suggesting that this antibody targets an epitope distant from the RBS. None of the isolated antibodies were able to inhibit agglutination of an H5N1 virus.

The mAb functions were further assessed by virus microneutralization (MN) assay (Figure 3D, right panel). All head and stem mAbs were able to neutralize the H1N1_pdm2009_ virus, with the head mAbs being more potent than the stem mAbs, as expected [51]. In agreement with the HAI data, the head mAb AF6H03 neutralized the H1N1_pdm2009_ and post-H1N1_pdm2009_ viruses. The stem mAb AF2S04 showed a broader neutralization compared to the head mAbs. AF2S04 potently neutralized pre-H1N1_pdm2009_, H1N1_pdm2009_ and post-H1N1_pdm2009_ viruses with similar potency as the stem-specific mAb CR6261. Interestingly, the trimer-only AF2T03 did not show any neutralization activity (>50 µg/mL). None of the mAbs, except CR6261, showed neutralization activity against the H5N1 virus.

## 4. Discussion

The humoral immune responses against the different domains of influenza A virus’ HA are not fully understood. A more comprehensive characterization of the specific binding patterns, the domains that are being targeted and the functional characteristics of antibodies formed after vaccination or infection will provide useful information to design and improve influenza virus vaccines.

Here, we report on immune assays involving three different probes representing the HA trimer, head and stem domains to analyze the overall antibody response directed against the influenza A virus’ HA. Remarkably, the antibodies present in the plasma of human donors were able to bind better the trimeric stem protein than to the monomeric head protein. These results contradict the general view that head antibodies are more abundant than stem antibodies, because of immuno-dominance of the head domain [52,53,54]. One caveat is that the vaccination and infection history of these donors is unknown and their blood was collected in 2019 while the probes were based on an H1N1_pdm2009_-virus strain originating approximately ten years before the collection date. One could speculate that the stem antibodies are boosted upon reinfection or vaccination with closely related H1N1 influenza A virus strains over the past ten years [55,56]. The head antibodies might be specific for the receptor binding domain of H1N1_pdm2009_ and more closely related viruses, as our isolated mAb AF6H03 suggests and, therefore, represented a smaller fraction in the plasma compared to the stem antibodies which are broadly reactive, as exemplified by our monoclonal antibody AF2S04. Expansion of our triple probe panel by including various virus strains and/or subtypes can help to further map the evolution of the head, stem and trimer-only specific antibody responses.

The differences between the relative contribution of head and stem antibody responses to the total response as measured in plasma by ELISA versus the staining pattern on B cells was striking. It is possible that differences in affinity and avidity in these assays play a role, as well the fact that three head domains are present on the HA-trimer protein versus one on the monomeric head protein. Although RBS-targeting mAbs 5J8 and 7B2 bound equally well to the monomeric head as to the full trimer in ELISA (Figure 1F, Figure S1), the difference in affinity and avidity of the B cells and antibodies to the binding of a multivalent or monomeric head domain may be different. This could potentially result in an underestimation of the (head) antibodies present in the plasma.

A major finding is that a large population of HA-trimer-specific B cells did not bind to the head nor stem probe. Soluble stem [53] or HA-trimer [50,57,58,59,60] probes have been used separately or combined, but combining the HA-trimer probe with monomeric head and trimeric stem probes as we have done here, clearly provides additional insight into the different HA-domains targeted by the B cells. It could be argued that using an HA-trimer probe in combination with trimeric-stem and monomeric head could lead to cross competition resulting in an underestimation of head and stem specificity of the response. However, this is contradicted by our observation that some of the head and stem specific responses show a high staining intensity (Figure 3A). We showed that the trimer-only population also dominates in a double-trimer stain (Figure S5). Furthermore, the frequency distribution of head, stem and trimer-only cells is similar when IgM+ cells were excluded for the analysis (Figure 2E and Table S2), indicating that the trimer-only population is not dominated by sticky-IgM. This suggests that, together with our characterized mAbs, only a minor fraction of the trimer-only population in our single trimer stain could be nonspecific for the HA-trimer. Including an IgD-marker in the flow cytometry panel can be a useful addition to exclude potential naïve B cells from the analysis. 

The majority of the trimer-only B cells potentially recognize domains on the trimer that is not accessible in the trimeric stem or monomeric head. These domains could include the interface between the head and stem domain, the fusion peptide, the interface between two head domains, the interface of three head domains at the trimer apex, or exposed domains when the trimer is in the open conformation. Several monoclonal antibodies have recently been isolated that target these domains [35,61,62,63,64,65] and combined with our data this suggests that previously underappreciated antibodies form a prominent component of the humoral response against influenza virus [61,62]. Our trimer-only mAb AF2T03 which targets a so far unknown domain that is not recapitulated by the stem nor head proteins lends further support for this supposition (Figure 3C). However, characterization of the trimer-only group needs further investigation to understand their target epitope and functionality.

In conclusion, using a novel set of influenza virus HA reagents we provide a comprehensive domain-oriented analysis of HA-specific responses at the antibody and the B cell level. Interestingly, we found a major population of B cells that only recognize the full HA-trimer, while head/RBS and stem domain targeting B cells form similar minority fractions. This major population needs to be characterized in more detail to establish the full spectrum of their fine specificities and functional characteristics. Moreover, it is important to understand how these antibodies are elicited. Whether these antibodies are a result of natural infection and/or vaccination and whether they are beneficial or better be avoided for a protective response towards influenza A virus infection remain open questions. These questions need to be addressed for a better understanding of influenza virus specific antibody responses and the development of a universal vaccine.

## Data Availability

Data will be made available upon request.

## Supplementary Materials

The following are available online at https://www.mdpi.com/article/10.3390/vaccines9070717/s1, Figure S1: Optical biosensor antibody binding patterns for HA-trimer, monomeric head and trimeric stem, Figure S2: Influence of different protein concentration coating on plasma and mAb responses, Figure S3: Flow cytometry gating strategy, Figure S4: Gating strategy and calculation of isotype frequency, Figure S5: Flow cytometry analysis of an alternative double HA-trimer stain of PBMCs of two healthy donors, Figure S6: Antibody expression and binding pattern in HEK293T cells, Figure S7: Excluding nonspecific tag-binding of AF2T03 mAb, Table S1: V-usage and CDR3 sequence and length of the isolated mAbs, Table S2: Frequency of each domain within the HA-trimer population for all B cell isotypes combined and separately.

## References

[1] Krammer F. (2019). The human antibody response to influenza A virus infection and vaccination. Nat. Rev. Immunol..

[2] Kosik I., Yewdell J.W. (2019). Influenza Hemagglutinin and Neuraminidase: Yin–Yang Proteins Coevolving to Thwart Immunity. Viruses.

[3] Altman M.O., Angeletti D., Yewdell J.W. (2018). Antibody Immunodominance: The Key to Understanding Influenza Virus Antigenic Drift. Viral Immunol..

[4] Zhuang Q., Wang S., Liu S., Hou G., Li J., Jiang W., Wang K., Peng C., Liu D., Guo A. (2019). Diversity and distribution of type A influenza viruses: An updated panorama analysis based on protein sequences. Virol. J..

[5] Smith D.J., Lapedes A.S., De Jong J.C., Bestebroer T.M., Rimmelzwaan G.F., Osterhaus A.D.M.E., Fouchier R.A.M. (2004). Mapping the Antigenic and Genetic Evolution of Influenza Virus. Science.

[6] Garten R.J., Davis C.T., Russell C.A., Shu B., Lindstrom S., Balish A., Sessions W.M., Xu X., Skepner E., Deyde V. (2009). Antigenic and Genetic Characteristics of Swine-Origin 2009 A(H1N1) Influenza Viruses Circulating in Humans. Science.

[7] Guthmiller J.J., Wilson P.C. (2018). Harnessing immune history to combat influenza viruses. Curr. Opin. Immunol..

[8] Goodwin K., Viboud C., Simonsen L. (2006). Antibody response to influenza vaccination in the elderly: A quantitative review. Vaccine.

[9] Lewnard J.A., Cobey S. (2018). Immune History and Influenza Vaccine Effectiveness. Vaccines.

[10] Andrews S.F., Huang Y., Kaur K., Popova L.I., Ho I.Y., Pauli N.T., Dunand C.J.H., Taylor W.M., Lim S., Huang M. (2015). Immune history profoundly affects broadly protective B cell responses to influenza. Sci. Transl. Med..

[11] Henry C., Palm A.-K.E., Utset H.A., Huang M., Ho I.Y., Zheng N.-Y., Fitzgerald T., Neu K.E., Chen Y.-Q., Krammer F. (2019). Monoclonal Antibody Responses after Recombinant Hemagglutinin Vaccine versus Subunit Inactivated Influenza Virus Vaccine: A Comparative Study. J. Virol..

[12] Dugan H.L., Guthmiller J.J., Arevalo P., Huang M., Chen Y.-Q., Neu K.E., Henry C., Zheng N.-Y., Lan L.Y.-L., Tepora M.E. (2020). Preexisting immunity shapes distinct antibody landscapes after influenza virus infection and vaccination in humans. Sci. Transl. Med..

[13] Gerhard W., Yewdell J.W., Frankel M.E., Webster R.G. (1981). Antigenic structure of influenza virus haemagglutinin defined by hybridoma antibodies. Nat. Cell Biol..

[14] Altman M., Bennink J.R., Yewdell J.W., Herrin B.R. (2015). Lamprey VLRB response to influenza virus supports universal rules of immunogenicity and antigenicity. eLife.

[15] Brandenburg B., Koudstaal W., Goudsmit J., Klaren V., Tang C., Bujny M.V., Korse H.J.W.M., Kwaks T., Otterstrom J.J., Juraszek J. (2013). Mechanisms of Hemagglutinin Targeted Influenza Virus Neutralization. PLoS ONE.

[16] Kirkpatrick E., Qiu X., Wilson P.C., Bahl J., Krammer F. (2018). The influenza virus hemagglutinin head evolves faster than the stalk domain. Sci. Rep..

[17] Petrova V.N., Russell C.A. (2018). The evolution of seasonal influenza viruses. Nat. Rev. Microbiol..

[18] de Jong N.M., Aartse A., van Gils M.J., Eggink D. (2020). Development of broadly reactive influenza vaccines by targeting the conserved regions of the hemagglutinin stem and head domains. Expert Rev. Vaccines.

[19] Angeletti D., Kosik I., Santos J., Yewdell W.T., Boudreau C.M., Mallajosyula V.V.A., Mankowski M.C., Chambers M., Prabhakaran M., Hickman H.D. (2019). Outflanking immunodominance to target subdominant broadly neutralizing epitopes. Proc. Natl. Acad. Sci. USA.

[20] Burke D.F., Smith D.J. (2014). A Recommended Numbering Scheme for Influenza A HA Subtypes. PLoS ONE.

[21] Yassine H.M., Boyington J.C., McTamney P.M., Wei C.-J., Kanekiyo M., Kong W.-P., Gallagher J.R., Wang L., Zhang Y., Joyce M.G. (2015). Hemagglutinin-stem nanoparticles generate heterosubtypic influenza protection. Nat. Med..

[22] Vanderven H.A., Wragg K., Ana-Sosa-Batiz F., Kristensen A.B., Jegaskanda S., Wheatley A.K., Wentworth D., Wines B.D., Hogarth P.M., Rockman S. (2018). Anti-Influenza Hyperimmune Immunoglobulin Enhances Fc-Functional Antibody Immunity During Human Influenza Infection. J. Infect. Dis..

[23] Martína J., Wharton S.A., Lin Y.P., Takemoto D.K., Skehel J.J., Wiley D.C., Steinhauer D.A. (1998). Studies of the Binding Properties of Influenza Hemagglutinin Receptor-Site Mutants. Virology.

[24] Binley J.M., Sanders R.W., Clas B., Schuelke N., Master A., Guo Y., Kajumo F., Anselma D.J., Maddon P.J., Olson W.C. (2000). A Recombinant Human Immunodeficiency Virus Type 1 Envelope Glycoprotein Complex Stabilized by an Intermolecular Disulfide Bond between the gp120 and gp41 Subunits Is an Antigenic Mimic of the Trimeric Virion-Associated Structure. J. Virol..

[25] Gibson D.G., Young L., Chuang R.-Y., Venter J.C., Hutchison C.A., Smith H.O. (2009). Enzymatic assembly of DNA molecules up to several hundred kilobases. Nat. Methods.

[26] Brouwer P.J.M., Caniels T.G., Van Der Straten K., Snitselaar J.L., Aldon Y., Bangaru S., Torres J.L., Okba N.M.A., Claireaux M., Kerster G. (2020). Potent neutralizing antibodies from COVID-19 patients define multiple targets of vulnerability. Science.

[27] Sliepen K., Han B.W., Bontjer I., Mooij P., Garces F., Behrens A.-J., Rantalainen K., Kumar S., Sarkar A., Brouwer P.J.M. (2019). Structure and immunogenicity of a stabilized HIV-1 envelope trimer based on a group-M consensus sequence. Nat. Commun..

[28] Tiller T., Meffre E., Yurasov S., Tsuiji M., Nussenzweig M.C., Wardemann H. (2008). Efficient generation of monoclonal antibodies from single human B cells by single cell RT-PCR and expression vector cloning. J. Immunol. Methods.

[29] Ho I.Y., Bunker J.J., Erickson S.A., Neu K.E., Huang M., Cortese M., Pulendran B., Wilson P.C. (2016). Refined protocol for generating monoclonal antibodies from single human and murine B cells. J. Immunol. Methods.

[30] Benckert J., Schmolka N., Kreschel C., Zoller M.J., Sturm A., Wiedenmann B., Wardemann H. (2011). The majority of intestinal IgA+ and IgG+ plasmablasts in the human gut are antigen-specific. J. Clin. Investig..

[31] Hong M., Lee P.S., Hoffman R.M.B., Zhu X., Krause J.C., Laursen N.S., Yoon S.-I., Song L., Tussey L., Crowe J. (2013). Antibody Recognition of the Pandemic H1N1 Influenza Virus Hemagglutinin Receptor Binding Site. J. Virol..

[32] Krause J.C., Tsibane T., Tumpey T.M., Huffman C.J., Basler C., Crowe J.E. (2011). A Broadly Neutralizing Human Monoclonal Antibody That Recognizes a Conserved, Novel Epitope on the Globular Head of the Influenza H1N1 Virus Hemagglutinin. J. Virol..

[33] Throsby M., van den Brink E., Jongeneelen M., Poon L.L.M., Alard P., Cornelissen L., Bakker A., Cox F., Van Deventer E., Guan Y. (2008). Heterosubtypic Neutralizing Monoclonal Antibodies Cross-Protective against H5N1 and H1N1 Recovered from Human IgM+ Memory B Cells. PLoS ONE.

[34] Ekiert D., Bhabha G., Elsliger M.-A., Friesen R.H.E., Jongeneelen M., Throsby M., Goudsmit J., Wilson I.A. (2009). Antibody Recognition of a Highly Conserved Influenza Virus Epitope. Science.

[35] Bangaru S., Lang S., Schotsaert M., VanderVen H.A., Zhu X., Kose N., Bombardi R., Finn J.A., Kent S.J., Gilchuk P. (2019). A Site of Vulnerability on the Influenza Virus Hemagglutinin Head Domain Trimer Interface. Cell.

[36] Ekiert D.C., Friesen R.H., Bhabha G., Kwaks T., Jongeneelen M., Yu W., Ophorst C., Cox F., Korse H.J., Brandenburg B. (2011). A highly conserved neutralizing epitope on group 2 influenza A viruses. Science.

[37] Friesen R.H., Lee P.S., Stoop E.J., Hoffman R.M., Ekiert D.C., Bhabha G., Yu W., Juraszek J., Koudstaal W., Jongeneelen M. (2014). A common solution to group 2 influenza virus neutralization. Proc. Natl. Acad. Sci. USA.

[38] Corti D., Voss J., Gamblin S.J., Codoni G., Macagno A., Jarrossay D., Vachieri S.G., Pinna D., Minola A., Vanzetta F. (2011). A Neutralizing Antibody Selected from Plasma Cells That Binds to Group 1 and Group 2 Influenza A Hemagglutinins. Science.

[39] Van Gils M.J., Van Den Kerkhof T.L., Ozorowski G., Cottrell C.A., Sok D., Pauthner M., Pallesen J., De Val N., Yasmeen A., De Taeye S.W. (2016). An HIV-1 antibody from an elite neutralizer implicates the fusion peptide as a site of vulnerability. Nat. Microbiol..

[40] Okuno Y., Isegawa Y., Sasao F., Ueda S. (1993). A common neutralizing epitope conserved between the hemagglutinins of influenza A virus H1 and H2 strains. J. Virol..

[41] Tan G.S., Krammer F., Eggink D., Kongchanagul A., Moran T.M., Palese P. (2012). A Pan-H1 Anti-Hemagglutinin Monoclonal Antibody with Potent Broad-Spectrum Efficacy In Vivo. J. Virol..

[42] Heaton N.S., Leyva-Grado V.H., Tan G.S., Eggink D., Hai R., Palese P. (2013). In Vivo Bioluminescent Imaging of Influenza A Virus Infection and Characterization of Novel Cross-Protective Monoclonal Antibodies. J. Virol..

[43] Hai R., Krammer F., Tan G.S., Pica N., Eggink D., Maamary J., Margine I., Albrecht R., Palese P. (2012). Influenza Viruses Expressing Chimeric Hemagglutinins: Globular Head and Stalk Domains Derived from Different Subtypes. J. Virol..

[44] Eggink D., Spronken M., van der Woude R., Buzink J., Broszeit F., McBride R., Pawestri H.A., Setiawaty V., Paulson J.C., Boons G.-J. (2020). Phenotypic Effects of Substitutions within the Receptor Binding Site of Highly Pathogenic Avian Influenza H5N1 Virus Observed during Human Infection. J. Virol..

[45] Reed L.J., Muench H. (1938). A simple method of estimating fifty per cent endpoints. Am. J. Epidemiol..

[46] WHO Global Influenza Surveillance Network: Manual for the Laboratory Diagnosis and Virological Surveillance of Influenza. https://apps.who.int/iris/bitstream/handle/10665/44518/9789241548090_eng.pdf?sequence=1.

[47] Thompson A.J., Cao L., Ma Y., Wang X., Diedrich J.K., Kikuchi C., Willis S., Worth C., McBride R., Yates J.R. (2020). Human Influenza Virus Hemagglutinins Contain Conserved Oligomannose N-Linked Glycans Allowing Potent Neutralization by Lectins. Cell Host Microbe.

[48] Cao L., Diedrich J.K., Ma Y., Wang N., Pauthner M., Park S.-K.R., Delahunty C.M., McLellan J., Burton D.R., Yates J.R. (2018). Global site-specific analysis of glycoprotein N-glycan processing. Nat. Protoc..

[49] Skehel J.J., Wiley D.C. (2000). Receptor Binding and Membrane Fusion in Virus Entry: The Influenza Hemagglutinin. Annu. Rev. Biochem..

[50] Whittle J.R.R., Wheatley A., Wu L., Lingwood D., Kanekiyo M., Ma S.S., Narpala S.R., Yassine H.M., Frank G.M., Yewdell J.W. (2014). Flow Cytometry Reveals that H5N1 Vaccination Elicits Cross-Reactive Stem-Directed Antibodies from Multiple Ig Heavy-Chain Lineages. J. Virol..

[51] Krause J.C., Crowe J.E. (2015). Committing the oldest sins in the newest kind of ways—Antibodies targeting the influenza virus type A hemagglutinin globular head. Antibodies Infect. Dis..

[52] Eggink D., Goff P.H., Palese P. (2013). Guiding the Immune Response against Influenza Virus Hemagglutinin toward the Conserved Stalk Domain by Hyperglycosylation of the Globular Head Domain. J. Virol..

[53] Tan H.-X., Jegaskanda S., Juno J.A., Esterbauer R., Wong J., Kelly H.G., Liu Y., Tilmanis D., Hurt A.C., Yewdell J.W. (2019). Subdominance and poor intrinsic immunogenicity limit humoral immunity targeting influenza HA stem. J. Clin. Investig..

[54] Wu N.C., Wilson I.A. (2019). Influenza Hemagglutinin Structures and Antibody Recognition. Cold Spring Harb. Perspect. Med..

[55] Sangster M.Y., Nguyen P.Q.T., Topham D.J. (2019). Role of Memory B Cells in Hemagglutinin-Specific Antibody Production Following Human Influenza A Virus Infection. Pathogens.

[56] Knight M., Changrob S., Li L., Wilson P.C. (2020). Imprinting, immunodominance, and other impediments to generating broad influenza immunity. Immunol. Rev..

[57] McCarthy K.R., Watanabe A., Kuraoka M., Do K.T., McGee C.E., Sempowski G.D., Kepler T.B., Schmidt A.G., Kelsoe G., Harrison S.C. (2018). Memory B Cells that Cross-React with Group 1 and Group 2 Influenza A Viruses Are Abundant in Adult Human Repertoires. Immunity.

[58] Watanabe A., McCarthy K.R., Kuraoka M., Schmidt A.G., Adachi Y., Onodera T., Tonouchi K., Caradonna T., Bajic G., Song S. (2019). Antibodies to a Conserved Influenza Head Interface Epitope Protect by an IgG Subtype-Dependent Mechanism. Cell.

[59] Koutsakos M., Sekiya T., Chua B.Y., Nguyen T.H.O., Wheatley A.K., Juno J.A., Ohno M., Nomura N., Ohara Y., Nishimura T. (2021). Immune profiling of influenza-specific B-and T-cell responses in macaques using flow cytometry-based assays. Immunol. Cell Biol..

[60] Fu Y., Zhang Z., Sheehan J., Avnir Y., Ridenour C., Sachnik T., Sun J., Hossain M.J., Chen L.-M., Zhu Q. (2016). A broadly neutralizing anti-influenza antibody reveals ongoing capacity of haemagglutinin-specific memory B cells to evolve. Nat. Commun..

[61] Lee J., Boutz D.R., Chromikova V., Joyce M.G., Vollmers C., Leung K., Horton A.P., DeKosky B.J., Lee C.-H., Lavinder J.J. (2016). Molecular-level analysis of the serum antibody repertoire in young adults before and after seasonal influenza vaccination. Nat. Med..

[62] Kuraoka M., Adachi Y., Takahashi Y. (2020). Hide and seek: Interplay between influenza viruses and B cells. Int. Immunol..

[63] Turner H.L., Pallesen J., Lang S., Bangaru S., Urata S., Li S., Cottrell C.A., Bowman C.A., Crowe J.E., Wilson I.A. (2019). Potent anti-influenza H7 human monoclonal antibody induces separation of hemagglutinin receptor-binding head domains. PLoS Biol..

[64] Dong J., Gilchuk I., Li S., Irving R., Goff M.T., Turner H.L., Ward A.B., Carnahan R.H., Crowe J.E. (2020). Anti–influenza H7 human antibody targets antigenic site in hemagglutinin head domain interface. J. Clin. Investig..

[65] Bangaru S., Zhang H., Gilchuk I.M., Voss T.G., Irving R.P., Gilchuk P., Matta P., Zhu X., Lang S., Nieusma T. (2018). A multifunctional human monoclonal neutralizing antibody that targets a unique conserved epitope on influenza HA. Nat. Commun..

