# Supplementary material for: Influenza A Virus Hemagglutinin Trimer, Head and Stem Proteins Identify and Quantify Different Hemagglutinin-Specific B Cell Subsets in Humans
Source: Vaccines (Basel). 2021 Jul 2;9(7):717. doi: 10.3390/vaccines9070717 (PMC8310015; doi:10.3390/vaccines9070717)
Supplement: Supplementary file 1 [file vaccines-09-00717-s001.zip › vaccines-1203217-supplementary.pdf]

Supplemental Figures corresponding to manuscript entitled:

Influenza A virus hemagglutinin trimer, head, and stem proteins identify and quantify different hemagglutinin-specific B cell subsets in humans

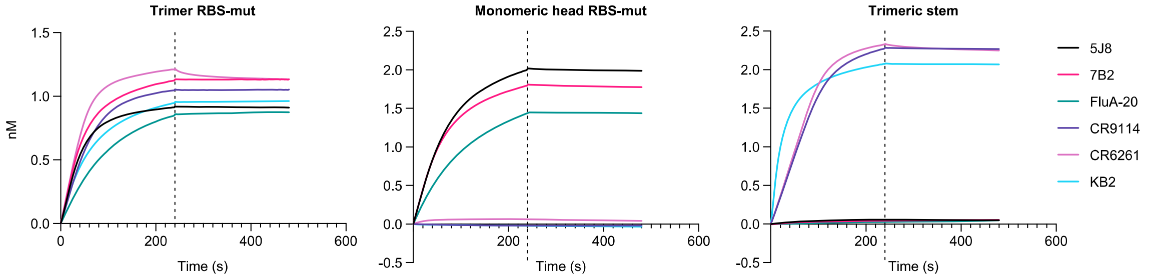

Figure S1: Optical biosensor antibody binding patterns for HA-trimer, monomeric head, and trimeric stem. Non-biotinylated HA-ttrimer, monomeric head, and trimeric stem proteins were immobilized on an optical biosensor until the threshold of 1 nM was reached. Several well-characterized head and stem mAbs were loaded in a concentration of 5 µg/mL. 5J8, 7B2, and FluA-20 are head specific mAbs and CR9114, CR6261, and KB2 are stem specific mAbs. Association and dissociation steps were set at 240 s for each mAb, indicated by the dotted line.

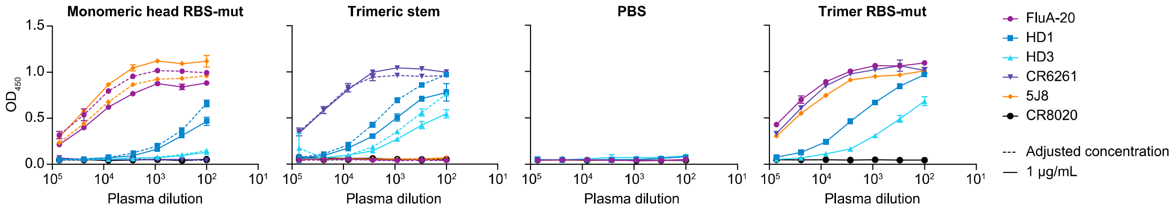

Figure S2: Influence of different protein concentration coating on plasma and mAb responses. Strep-Tactin XT ELISA plates coated with HA-trimer (1.0 µg/mL), monomeric head (at 1.0 or 0.58 µg/mL), and trimeric stem (1.0 or 0.44 µg/mL) for coating corrected by concentration or molecular weight and possible binding sites of the antibodies to the protein. The solid line represents the 1 µg/mL and the dotted line shows the binding of plasma and mAbs with the adjusted coating to molecular weight. mAb CR8020 was used as a negative control.

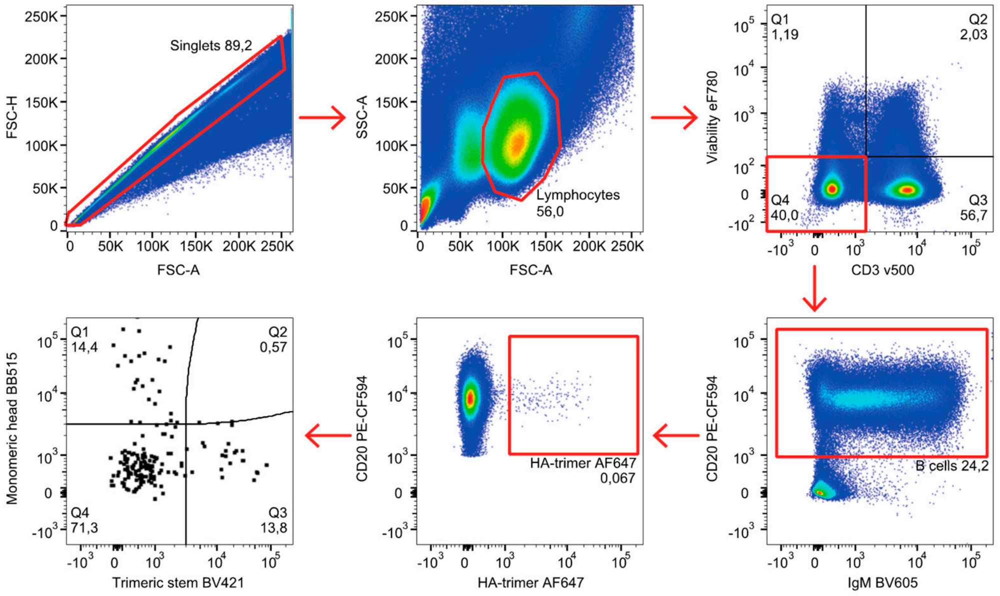

Figure S3: Flow cytometry gating strategy. Flow cytometry gating strategy example of HA-trimer specific B cells in healthy donors. The gating was performed by selecting cells using the following parameters: singlets, lymphocytes, CD3- and viability- (excluding dead cells and T cells), CD20+ and IgM+/- (B cells), and HA-trimer+. The lower left panel reveals the head and stem specific B cells among the HA-trimer+ cells.

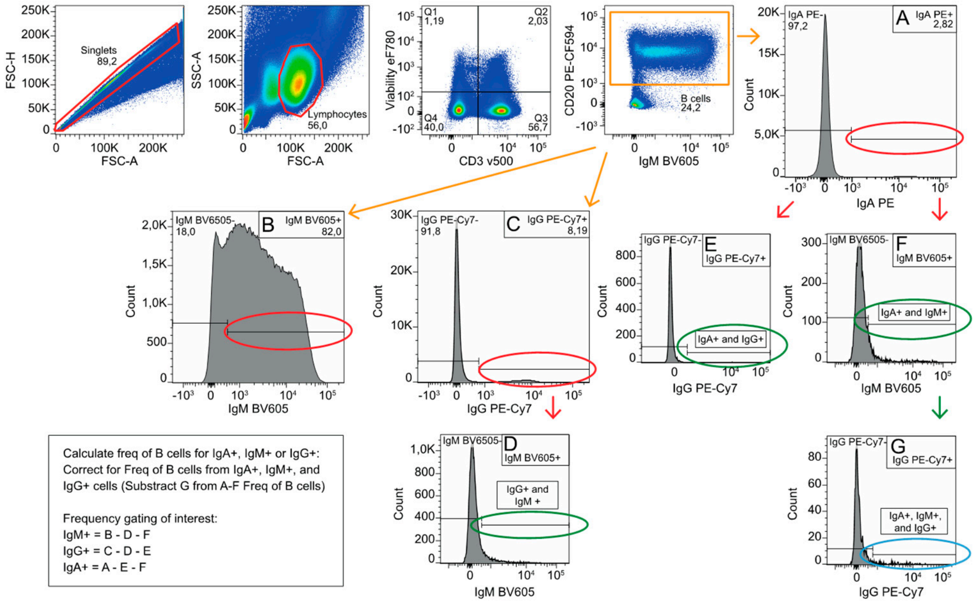

Figure S4: Gating strategy and calculation of isotype frequency. The frequencies of the gates were taken for the population of interest, e.g. B cells (yellow). First a correction was made for cells that were positive for all three isotypes (IgA+, IgM+, and IgG+, in G, blue). Next, the cells that had two isotypes (D, E, and F in green) were excluded to obtain the percentage of B cells per isotype, e.g. IgA, IgM, or IgG (A, B, and C in red). Unclassified B cells were double/triple positive or negative for all isotypes. The same strategy was used for the isotypes of HA-trimer positive cells, and for monomeric head, trimeric stem, and trimer-only cells.

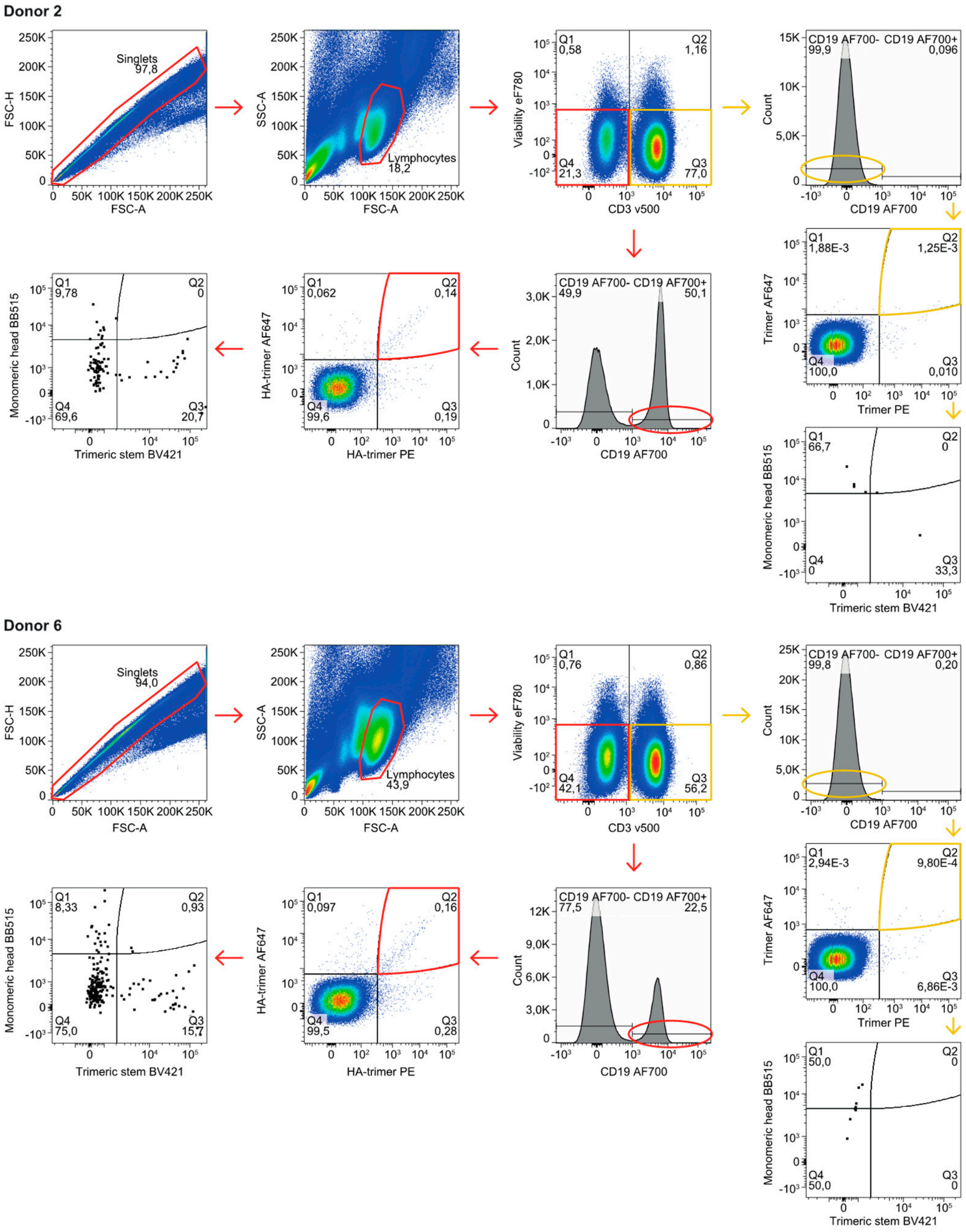

Figure S5: Flow cytometry analysis of an alternative double HA-trimer stain of PBMCs of two healthy donors. The gating (in red highlighted) was performed by selecting the following populations and parameters: Singlets, lymphocytes, CD3- (UCHT1, BD Horizon), viability- (Invitrogen), CD19+ (HIB19, BioLegend), and HA-trimer+/+. The head or stem binding cells can be observed within this double positive HA-trimer population. Alternative gating (in yellow highlighted) was performed on singlets, lymphocytes, CD3+, viability-, CD19-, and HA-trimer +/+. This gating was performed to evaluate non-specific binding of the HA probes to sialic acids on CD3+ cells.

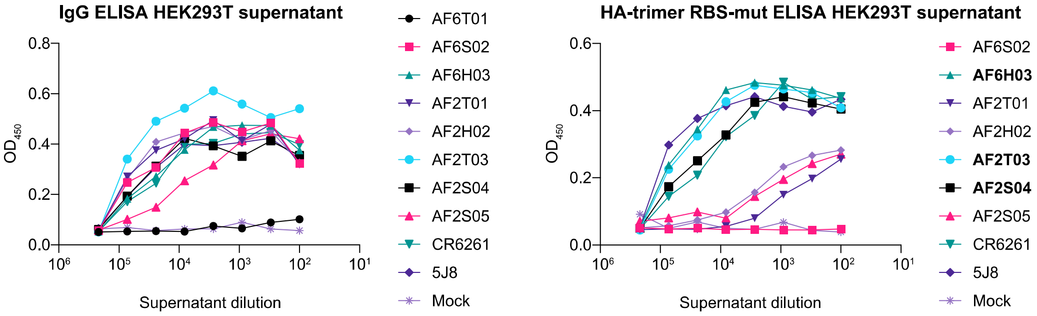

Figure S6: Antibody expression and binding pattern in HEK293T cells. HEK293T cells were co-transfected with expression vectors for the heavy and light chain and the supernatant was tested in an IgG ELISA (left) and a Strep-Tactin XT ELISA coated with HA-trimer RBS-mut (right). AF6T01 was negative in the IgG ELISA and therefore excluded from the HA-trimer ELISA. The three antibodies selected for further analysis are highlighted (AF6H03, AF2T03, and AF2S04).

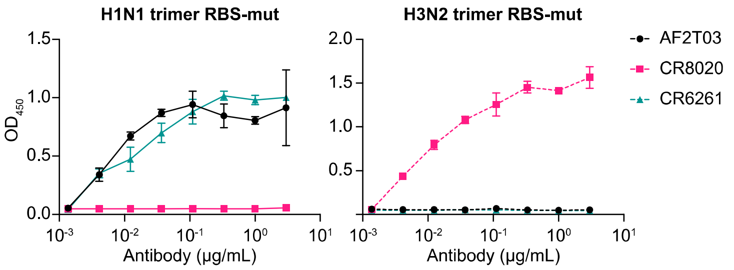

Figure S7: Excluding nonspecific tag-binding of AF2T03 mAb. Biotinylated HA-trimer RBS-mut from H3N2 (A/Netherlands/213/2003) and H1N1 (A/Netherlands/602/2009) were coated on a Strep-Tactin XT ELISA, The binding of mAbs AF2T03, CR6261, and CR8020 was evaluated in duplicates to each of the proteins.

Table S1: V-usage and CDR3 sequence and length of the isolated mAbs. V-usage and CDR3 sequence and length obtained from IMGT/V-QUEST database (program version: 3.5.25, reference directory release: 202118-1

Antibody name	V-usage	CDR3 amino acids	CDR3 length
AF2T03	IGHV3-30	AKDVSIAARYFDD	13
	IGKV3-15	LQFNKWPPLT	10
AF2S04	IGHV1-69	ARDRDDSTFGL	11
	IGKV1-5	QQSNTFSRT	9
AF6H03	IGHV3-30-3	ARGGGVNVDTVMCNYFDY	18
	IGKV1-33	QQYDNLPIT	9

Table S2. Frequency of each domain within the HA-trimer population for all B cell isotypes combined and separately. Cells with more than one isotype were not excluded, mean of six HDs.

Binding domain	All isotypes (Fig. 2E)	IgM-	IgM+	IgA+	IgG+
Head	14.7	16.3	13.1	11.7	21.9
Head+stem	3.5	2.3	4.8	0.7	4.1
Stem	14.3	18.6	12.6	34.7	25.7
Trimer-only	67.5	62.8	69.5	52.9	48.3
